# Glucose Metabolic Changes in the Brain and Muscles of Patients with Nonspecific Neck Pain Treated by Spinal Manipulation Therapy: A [^18^F]FDG PET Study

**DOI:** 10.1155/2017/4345703

**Published:** 2017-01-12

**Authors:** Akie Inami, Takeshi Ogura, Shoichi Watanuki, Md. Mehedi Masud, Katsuhiko Shibuya, Masayasu Miyake, Rin Matsuda, Kotaro Hiraoka, Masatoshi Itoh, Arlan W. Fuhr, Kazuhiko Yanai, Manabu Tashiro

**Affiliations:** ^1^Division of Cyclotron Nuclear Medicine, Cyclotron and Radioisotope Center, Tohoku University, Sendai, Japan; ^2^Japan Chiropractic Doctor College, Sendai, Japan; ^3^Department of Nuclear Medicine, United Hospital, Dhaka, Bangladesh; ^4^Sendai Medical Imaging Clinic, Sendai, Japan; ^5^Activator Methods International, Ltd., Phoenix, AZ, USA; ^6^Department of Pharmacology, Graduate School of Medicine, Tohoku University, Sendai, Japan

## Abstract

*Objective*. The aim of this study was to investigate changes in brain and muscle glucose metabolism that are not yet known, using positron emission tomography with [^18^F]fluorodeoxyglucose ([^18^F]FDG PET).* Methods*. Twenty-one male volunteers were recruited for the present study. [^18^F]FDG PET scanning was performed twice on each subject: once after the spinal manipulation therapy (SMT) intervention (treatment condition) and once after resting (control condition). We performed the SMT intervention using an adjustment device. Glucose metabolism of the brain and skeletal muscles was measured and compared between the two conditions. In addition, we measured salivary amylase level as an index of autonomic nervous system (ANS) activity, as well as muscle tension and subjective pain intensity in each subject.* Results*. Changes in brain activity after SMT included activation of the dorsal anterior cingulate cortex, cerebellar vermis, and somatosensory association cortex and deactivation of the prefrontal cortex and temporal sites. Glucose uptake in skeletal muscles showed a trend toward decreased metabolism after SMT, although the difference was not significant. Other measurements indicated relaxation of cervical muscle tension, decrease in salivary amylase level (suppression of sympathetic nerve activity), and pain relief after SMT.* Conclusion*. Brain processing after SMT may lead to physiological relaxation via a decrease in sympathetic nerve activity.

## 1. Introduction

Spinal manipulation therapy (SMT), which is performed by healthcare practitioners such as chiropractors, osteopathic physicians, and physiotherapists, has been applied mainly to musculoskeletal problems such as neck pain or low back pain. Many investigators have performed various experiments to elucidate the mechanism underlying the clinical effects of SMT. The earliest studies analyzed the magnitude of the force applied to the vertebrae and the movement of vertebrae during SMT, with the forces exerted by the practitioner quantified using a flexible pressure mat [1, 2]. The rotation and relative movement of vertebrae after treatment using a mechanical adjusting device (activator adjusting instrument, AAI) [3] have also been reported [4–8]. Previous studies have suggested that SMT has beneficial clinical effects, including pain relief [9] and reduction of blood pressure [10, 11]. It is thought that biomechanical input by SMT could generate a physical response or reflex [12]; however, the mechanism of these clinical effects induced by SMT is still unknown.

In recent years, the findings of brain activation studies using imaging modalities such as functional magnetic resonance imaging, near-infrared spectroscopy, and positron emission tomography (PET) have contributed to advances in brain science [13]. Such studies are able to enhance our understanding of the neurophysiological effects of specific physical/psychological tasks by detecting associated activation and deactivation of brain regions. PET has also been used to elucidate the metabolic changes that alternative therapies induce in living tissues; for example, an ^18^F-labeled glucose analog has been used to study cerebral metabolic changes after acupuncture [14] and aromatherapy [15], and [^15^O]H_2_O has been used to assess changes in cerebral blood flow during massage [16]. Lystad and Pollard have noted the usefulness of neuroimaging techniques for gaining a better understanding of the neurophysiological effects of SMT [17].

The [^18^F]-labeled glucose analog, fluorodeoxyglucose ([^18^F]FDG), is thought to be able to visualize the energy metabolism of various tissues such as brain and muscles in vivo. One of important advantages of [^18^F]FDG PET is that we can simultaneously measure the energy metabolism of brain and muscles. We have hypothesized that clinical effects such as muscle tension relaxation and pain relief are mediated by the regional brain activity induced by SMT. We have also expected any changes in muscular energy consumption in treatment condition. In addition, we have also expected significant correlation between the regional brain activity and the muscular energy consumption.

Previously, we reported preliminary results of our study to examine our initial hypothesis that a [^18^F]FDG PET can visualize brain metabolic changes induced by SMT [18]. In this paper, we report our conclusive results on this issue. In addition, the present work tries to examine our additional hypothesis that the [^18^F]FDG PET can visualize muscular metabolic changes induced by SMT, as well as their association with the regional brain metabolism.

## 2. Subjects and Methods

We recruited 21 male subjects (mean age ± SD: 26.4 ± 5.9 years) with cervical pain and shoulder stiffness but without abnormalities in neck-to-shoulder MR images and without history of any treatments prior to the present study. We performed MRI examination of the neck to the shoulder area on all participants; the resulting MR images were used as a reference for the anatomical locations of cervical muscles in PET images. Female subjects were not included in this study because of physiological fluctuations due to the menstrual cycle [19–23].

**Figure 1:**
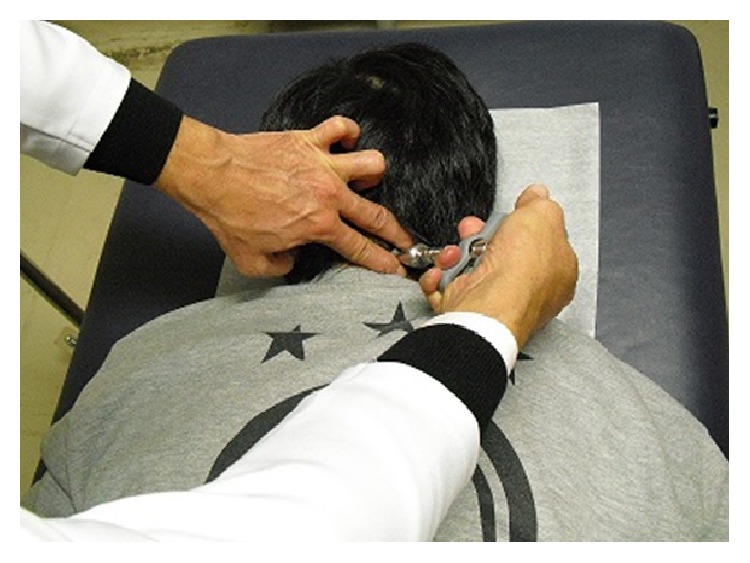
The location of cervical adjustment by the activator adjusting instrument (AAI) in the treatment condition. Spinal manipulation therapy (SMT) by AAI was performed by contact on the joints and did not include muscle massage. SMT on all subjects was performed by the same chiropractor, who was an advanced practitioner of Activator Methods.

SMT was applied using an AAI, Activator II (Activator Methods International, Ltd., Phoenix, USA), in accordance with the Activator Methods (AM) basic scan protocol [24, 25]. We utilized the AAI to apply impulses to specific vertebrae or joints (Figure 1). SMT was performed on the subject in a prone position without movements such as cervical rotation, lateral flexion, and extension, in order to prevent the muscular [^18^F]FDG uptake due to muscle contractions during the therapeutic procedure. SMT was carried out on the whole spine, the scapulae, the ilium, and the sacrum, as necessary for each subject. The mean number of SMT-adjusted sites was 8 per subject.

**Figure 2:**
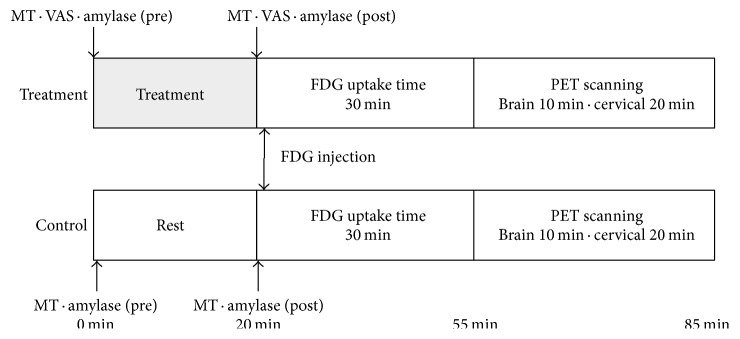
Diagram of the study protocol. Half of the subjects were randomly assigned to be scanned first in the resting condition and then in the treatment condition; the other half were scanned in the reverse order. The intensity of subjective pain was evaluated before and after spinal manipulation therapy only in the treatment condition. Muscle stiffness and salivary amylase were measured before and after the treatment or resting period. MT = muscle tension, VAS = visual analog scale.

PET scanning was performed twice on each subject according to the protocol given in Figure 2. The interval between the two scans ranged from 7 to 70 days (mean interval ± SD: 23 ± 15 days). The subjects received SMT intervention which lasted for approximately 20 minutes including a diagnostic procedure. Soon after the treatment, [^18^F]FDG-containing saline solution was injected to the subject through the left cubital vein (mean ± SD: 47.0 ± 8.9 MBq) in quiet room with a dim light next door to the treatment room in the same building. On the other scan day, [^18^F]FDG was injected to the subject after a 20-minute-long resting phase instead of SMT intervention. The subjects in both conditions were asked to sit in a relaxed position with their eyes closed for 30 minutes before PET scanning. The brain scan and the scan on the neck to the shoulder area of the subject were initiated after 30 minutes of [^18^F]FDG injection, utilizing a PET scanner, SET2400W (Shimadzu, Inc., Kyoto, Japan). The PET scanning covered the entire brain in one scan, taking 10 minutes for the emission scan and another 5 minutes for the transmission scan for tissue attenuation correction. Scanning from the neck to the shoulder area in one scan took 5 minutes for the emission scan and another 5 minutes for the transmission scan for tissue attenuation correction. Images were acquired with a 128 × 128 matrix and reconstructed using Fourier rebinning and Ordered Subset Expectation Maximization Algorithm [18]. The intensity of subjective pain was evaluated using a 0–10 visual analog scale (VAS) before and after SMT in the treatment condition. VAS evaluation was not done in the resting condition except for in 9 subjects. Cervical muscle tension was measured bilaterally at the superior part of the trapezius muscle using a tissue hardness meter (Muscle Meter PEK-1, Imoto Inc., Kyoto, Japan); the mean value of three measurements was recorded. Additionally, salivary amylase levels were measured for each subject using an amylase monitor (Nipro Inc., Osaka, Japan) to evaluate changes in autonomic nervous system (ANS) function. Figure 2 shows the measurement points. Further details of the study protocol are described in our previous report [18]. The whole protocol was approved by the Ethics Committee of Tohoku University Graduate School of Medicine, Sendai, Japan (number 2008-115).

For data analysis, differences in values before and after treatment and between the control and treatment conditions were compared using paired *t*-tests for all measurements except for PET data. Brain PET images were analyzed using the voxel-wise statistical analysis software package Statistical Parametric Mapping 8 (SPM8; Functional Imaging Laboratory, London, UK) in order to identify regional glucose metabolic changes [26, 27]. An FDG brain template distributed by Montreal Neurological Institute (McGill University, Montreal, Canada) [28] was used for anatomical standardization (spatial normalization) of the PET images. Each voxel had dimensions of 2 × 2 × 2 mm in the normalized image. The normalized data were smoothed using an isotropic Gaussian kernel of 12 mm (for the *x*-, *y*-, and *z*-axes) to increase the signal-to-noise ratio by suppressing high-frequency noise. The threshold for the statistical significance of the voxel value height in the present study was set at *p* < 0.05 with correction for multiple comparisons (family-wise error correction), while our previous study applied the compromised threshold of *p* < 0.001 without correction. Voxel values of the PET images were compared between the resting and treatment conditions using a paired *t*-test.

PET images of the neck and shoulder regions were coregistered to the MR images of the same subject; regions of interest (ROIs) for cervical muscles were then manually drawn on the PET images using Dr. View software (Version 2.0, AJS, Tokyo, Japan), using the MR images as references. ROIs were drawn on the trapezius muscle at C7-T1 levels bilaterally, the splenius muscles, the semispinalis muscles, the elevator scapular muscles, and the trapezius muscles at C6-C7 levels bilaterally. The standardized uptake value (SUV) for each muscle was calculated using the following formula:

(1) SUV=tissue  radioactivity  concentrationBq/g×body  weightginjected  activityBq.

SUVs were statistically examined using a paired *t*-test to compare muscle metabolism after SMT and after resting. In addition, the authors searched for specific brain regions associated with muscular energy consumption by applying linear correlation analysis using SUV of each muscle studied here as a parameter in SPM8.

## 3. Results

**Figure 3:**
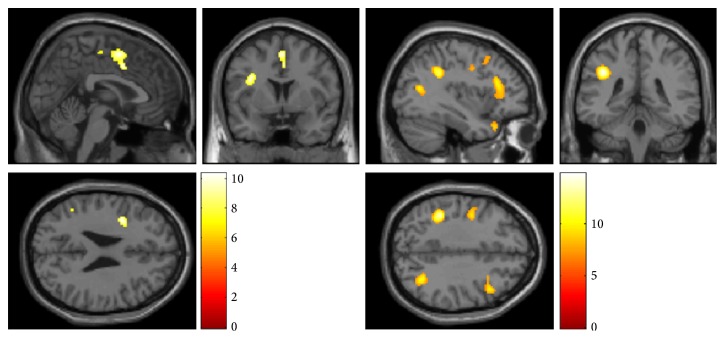
Regional activation (left) and deactivation (right) after spinal manipulation therapy (SMT) using an activator adjusting instrument. Glucose metabolism is increased in regions including the anterior cingulate cortex and cerebellar vermis but is relatively reduced in many sites, including the prefrontal cortex, after SMT. The voxel height threshold is *p* < 0.05, corrected for multiple comparisons; the extent threshold is 10 voxels minimum.

**Figure 4:**
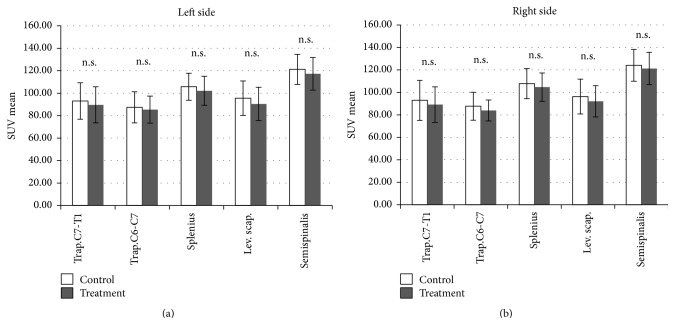
Results of positron emission tomography analysis of cervical muscles (paired *t*-test). The error bars represent standard deviations. The results indicate a trend toward reduction of mean standardized uptake value (SUV) after SMT; however, the difference is not statistically significant. Trap., trapezius muscle; Splenius, splenius muscles; Lev. Scap., levator scapulae; Semispinalis, semispinalis muscles; C7-T1, between the seventh cervical spine and the first thoracic spine; C6-C7, between the sixth cervical spine and the seventh cervical spine.

The SMT-associated regional brain metabolic changes (activation and deactivation) detected by the SPM8 analysis are shown in Table 1. Statistically significant areas were overlaid on the standard MRI brain template images (Figure 3). PET analysis of the cervical muscles showed a trend toward reduced metabolism (SUV) after SMT compared with the control condition; however, these changes were not statistically significant (Figure 4). In addition, no meaningful correlation was detected between muscular SUV and regional brain activity.

**Figure 5:**
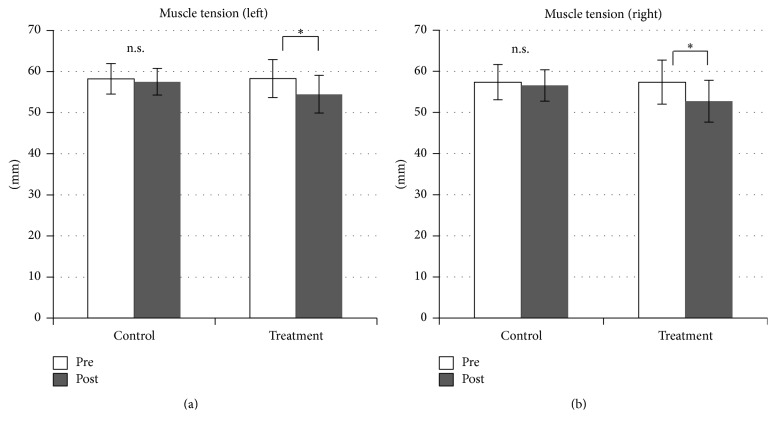
Muscle tension is significantly reduced after spinal manipulation therapy. ^*∗*^*p* < 0.0001.

**Figure 6:**
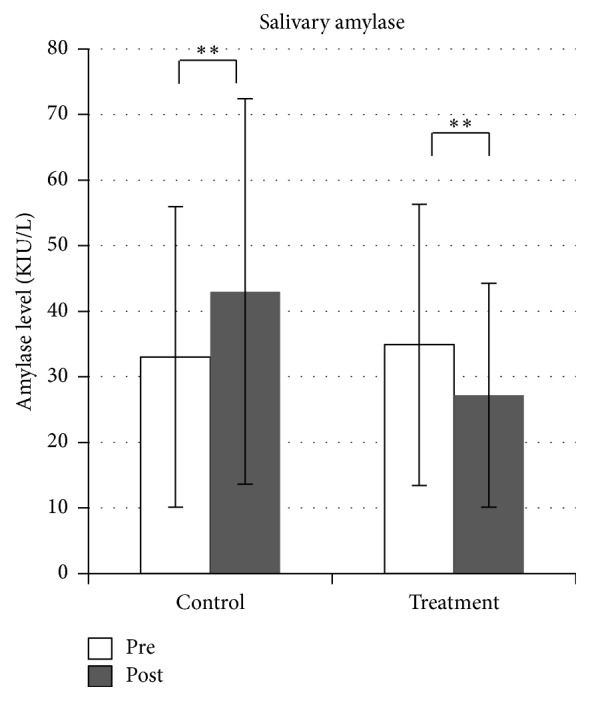
Changes in salivary amylase level. Salivary amylase level is reduced after spinal manipulation therapy but increased in the control condition. ^*∗∗*^*p* < 0.05.

**Figure 7:**
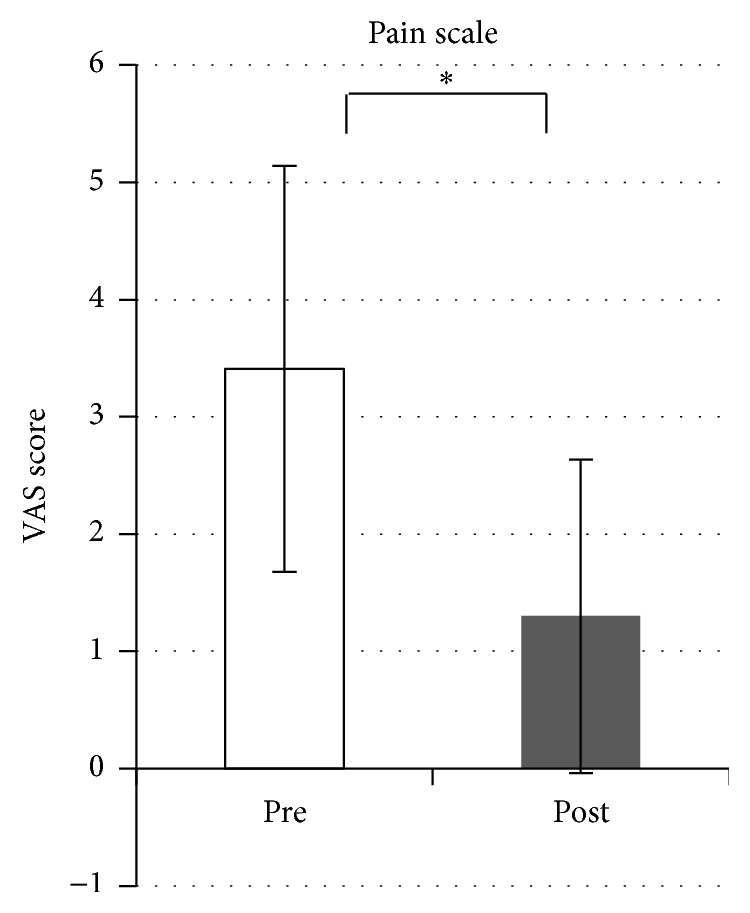
Changes in subjective pain in the treatment condition. The pain scale score is significantly decreased after spinal manipulation therapy. ^*∗*^*p* < 0.0001.

In contrast, cervical muscle tension was significantly reduced bilaterally after SMT (*p* < 0.0001 for both sides, Figure 5). Salivary amylase level decreased significantly after SMT (*p* = 0.022) but increased significantly in the control condition (*p* = 0.011, Figure 6). Comparisons of VAS pain scores in the treatment condition revealed a significant decrease after SMT (*p* < 0.0001, Figure 7), while the comparison in the control condition showed nonsignificant difference (*n* = 9).

## 4. Discussion

The findings of the present study demonstrate how stimuli to the mechanoreceptors of the joints and skin during SMT are processed in the brain. Injected [^18^F]FDG was absorbed into activated brain regions and visualized by PET. We observed multiple changes in brain activity after SMT.

SPM8 analyzes approximately half a million voxels of brain volume data simultaneously. Correction for multiple comparisons is therefore indispensable, making the statistical threshold extremely high. Since many studies have failed to detect significant differences in voxels after correction for multiple comparisons, the SPM8 development team proposed the use of a compromised threshold for voxel height (*p* < 0.001) combined with a voxel extent threshold for the size of each voxel cluster (e.g., 10 voxels minimum), as used in our previous report [26, 27]. This technique has been useful for practical purposes but is prone to Type-1 errors. The significant voxel clusters that survived correction for multiple comparisons in the present work may therefore indicate more robust and reliable findings than those in our preliminary report [18]; we believe these results are worthy of reporting as conclusive findings, despite the fact that the intensity of the observed brain responses to SMT intervention was initially estimated to be much weaker.

As for regional brain metabolic changes after the SMT intervention, activation (increased metabolism) was detected in the dACC (Brodmann area [BA] 32), cerebellar vermis (CV), and somatosensory association cortex, and regional deactivation (decreased metabolism) was detected in regions including the prefrontal cortex (PFC) and temporal sites. Involvement of the ACC in cognitive and emotional processes has been recognized since Papez mentioned the idea in 1937 [29–31], and this area is also involved in placebo and opioid analgesia [32]. Some brain activation studies have also demonstrated activation of the ACC in response to pleasant or unpleasant stimuli such as massages or the olfactory stimulus of isovaleric acid, respectively [33–35]. The ACC is part of network that carries out cognitive processing based on individual factors such as experiences or emotions while making contact with the other regions in the network, such as the limbic system and cortex [32, 36]. Recently, specific features of the dACC functions and their connectivity to other brain regions are not fully elucidated yet, though they have been revealed little by little. For example, dACC is involved in cognitive functions, motivation, and reward-based decision-making as a part of the network including the CV and prefrontal cortex [37]. The neuronal activity changes in dACC and CV were detected in the present study.

The CV receives somatic sensory information from the spinal cord and via the vestibulospinal tract or reticular nuclei of the brainstem through the spinal cord, connecting indirectly or directly with motor cells on the ventral horn. These systems control involuntary muscular tension and reflexes. Our results suggest that stimulation of joints during SMT induced relaxation of reflexive muscle tension. The cerebellum is also thought to have a functional role as an integrator of multiple effector systems, including affective processing, pain modulation, and sensorimotor processing [38]. Recently, many studies have reported roles for the cerebellum in nonmotor functions [39, 40]. A study by Sacchetti et al. showed that the CV is activated during mental recall of emotional personal episodes in humans [39]. Lou et al. also reported that the CV, ACC, and some regions of the PFC are activated during relaxation mediation in yoga [41]. However, it is important to note that the PFC was deactivated after SMT in our study. Interestingly, Critchley et al. found that the ACC/dACC (BA24/32) and CV are specifically activated during biofeedback therapy [42], a technique for controlling one's tension to generate a state of relaxation. The activated areas in the present study are similar to those activated during biofeedback relaxation, indicating that the state of the brain after SMT may be similar to that induced by biofeedback therapy. Furthermore, our assessment of body responses in this study showed relaxation of muscle tension and decreased salivary amylase levels—phenomena that are associated with reduced sympathetic nerve activity. Salivary *α*-amylase levels correspond to plasma norepinephrine levels and are utilized as an accessible measure of sympathetic nervous reactivity in stress research, with lower levels indicating lower activity [43–45].

Ouchi et al. suggested that the comfortable sensation generated by back massage may be related to increased regional cerebral blood flow in the posterior brain—specifically, in the precuneus [16]. SMT stimuli to the joints may be processed differently from those of muscle massage, resulting in decreased sympathetic nerve activity. On the other hand, certain cervical muscles showed a tendency toward decreased glucose metabolism after SMT, although the difference was not statistically significant (Figure 4). Increasing the number of study subjects may increase the significance of this finding. The underlying mechanism of reduced muscular glucose uptake is not yet understood; however, an animal study suggests the involvement of sympathetic nerve activity [46]. Glucose uptake in skeletal muscles may thus be influenced directly or indirectly by the ANS.

Although the mechanism of muscle relaxation is still unknown, we hypothesize the involvement of (a) autonomic nervous activity and (b) improvement of the range of joint movement [47]. In the present study, we observed that SMT stimulus induced physical responses such as muscle tension relaxation, pain relief, and reduced amylase secretion. These changes may be associated with neural processing in the dACC and CV. Neural inputs evoked by SMT stimuli via various receptors in muscles, tendons, and joints may ascend to the somatosensory areas of the brain through the medial lemniscal system. The response signal descending from the brain may then adjust the postural muscles accordingly.

Muscular SUV, an index of energy consumption of skeletal muscles, did not show significant difference between the control and treatment conditions, while muscle tension did show significant difference. In addition, we could not detect meaningful correlation between the SUV of muscular glucose consumption and the regional brain glucose consumption.

As is the case with every study, our methodology is not without limitations. Ideally, the present subjects would have undergone PET scanning before and after the SMT intervention in both the control and treatment conditions. However, this protocol would have assigned 4 PET scans to each subject, resulting in unreasonably high radiation exposure for this kind of study. Furthermore, it is hard to perform serial PET scans within 1 or 2 hours of each other because of the relatively long physical half-life of the [^18^F] nuclide (110 minutes). In addition, the number of subjects (*n* = 21) was still relatively small for a clinical study; however, we define the present results as conclusive ones in order to minimize radiation exposure of the subjects, who were healthy other than their neck and shoulder symptoms and who are considered to be part of the general public. Previously, we reported preliminary findings with a sample size of 12; however, the study results were only at threshold level [18], and these results seemed to be prone to Type-1 errors because they were based on a compromised statistical examination without correction for multiple comparisons. On the other hand, no voxels survived with a standard statistical examination including correction for multiple comparisons, suggesting that these negative results may be prone to Type-2 errors. By raising the sample size to 21, now we are able to obtain robust results that survived even after correction for multiple comparisons. Thus, the authors believe the present results are more reliable as an evidence for further discussion on clinical effects of SMT interventions while our preliminary report was useful as a “proof of concept” study.

## 5. Conclusion

In summary, we observed metabolic changes in the brain and skeletal muscles, as well as reductions in subjective pain, muscle tension, and salivary amylase, after SMT intervention. These results may be associated with reduced sympathetic nerve activity, suggesting that SMT induces a kind of relaxation similar to that achieved by biofeedback. The brain response to SMT may reflect the psychophysiological relaxation that accompanies reduced sympathetic nerve activity.

## Figures and Tables

**Table 1:** Brain metabolic changes associated with the spinal manipulation therapy intervention.

Anatomical region	Coordinates *x*, *y*, and *z* (mm)	Brodmann area	Cluster equiv.	Voxel *Z* score
Activation				
Broca's area	−34, 6, 28	44	110	5.84
ACC	2, 8, 40	32	228	5.72
SSAC	16, −26, 48	5	114	5.65
Wernicke's area	46, −48, 20	22	30	5.52
VAC	−6, −88, 38	19	31	5.32
CV	6, −62, −4	—	14	5.28
VC (V2)	24, −80, 6	18	17	5.24
Deactivation				
IPL	−40, −40, 34	39/40	286	6.74
FP	−2, 68, −8	10	160	6.66
IFG PT	40, 28, 16	45	117	6.53
PSMA	30, 14, 44	6	348	6.34
PMC (M1)	−28, −18, 56	4	45	6.12
FEF/dl-PFC	−30, 12, 44	8/9	157	5.93
dl-PFC	−38, 26, 24	46	309	5.70
AG/FG	−40, −60, 8	39/37	177	5.58
ITG	−70, −22, −20	20	20	5.66
TP	40, 22, −44	38	12	5.64
CV (V1)	−14, −66, 24	17	46	5.29

Brain metabolic changes detected by SPM8 are presented (voxel height threshold *p* < 0.05 with corrections for multiple comparisons, extent threshold 10 voxels minimum). The statistical significance of regional metabolic changes is given as *Z* scores [(Mean_treatment_ − Mean_control_)/SD_control_].

ACC, anterior cingulate cortex; SSAC, somatosensory association cortex; VAC, visual association cortex; CV, cerebellar vermis; VC, visual cortex; IPL, inferior parietal lobule; FP, frontal pole; IFG, inferior frontal gyrus; PT, pars triangularis; PSMA, premotor area/supplementary motor area; PMC, primary motor cortex; FEF, frontal eye field; dl-PFC, dorsolateral prefrontal cortex; AG, angular gyrus; FC, fusiform gyrus; ITG, inferior temporal gyrus; TP, temporal pole.
